# Masseter Muscle Thickness and Mandibular Trabecular Complexity: A Cross-Sectional Study

**DOI:** 10.4317/jced.64184

**Published:** 2026-06-29

**Authors:** Diego De Nordenflycht, Camila Antúnez

**Affiliations:** 1 DDS, MSc. Faculty of Dentistry, Universidad Andres Bello, Viña del Mar, Chile; 2 DDS. Private practice. Viña del Mar, Chile

## Abstract

**Background:**

To evaluate the association between masseter muscle thickness and mandibular trabecular complexity in subjects with and without myogenous temporomandibular disorders (TMDs).

**Materials and Methods:**

A cross-sectional study was conducted including 77 participants (41 controls and 36 with TMD). Masseter muscle thickness was measured using ultrasonography, and fractal analysis was performed on panoramic radiographs. Associations were assessed using Spearman correlation and linear regression models. Multivariable analyses were adjusted for age, sex, and diagnostic group.

**Results:**

A weak positive correlation was observed between masseter thickness and fractal dimension ( = 0.255, p = 0.025). In unadjusted regression, masseter thickness was significantly associated with fractal values (p = 0.019). However, this association was attenuated and no longer statistically significant after adjustment for age, sex, and diagnostic group (p = 0.091). Sex was significantly associated with masseter thickness (p &lt; 0.001) but not with fractal dimension. In contrast, TMD was independently associated with higher fractal dimension values (p = 0.011). No significant interaction between masseter thickness and diagnostic group was observed.

**Conclusions:**

Masseter muscle thickness shows a weak association with mandibular trabecular complexity that varies according to demographic factors. In contrast, TMD is independently associated with higher fractal dimension values, suggesting that mandibular bone microarchitecture is associated with complex functional and clinical influences beyond muscle morphology alone.

## Introduction

Bones continuously adapt their cortical and trabecular architecture in response to mechanical loading (1). According to the mechanostat hypothesis, the microdeformation of the extracellular matrix triggers cellular responses that result in either bone modeling to increase mass or resorption to eliminate metabolically expensive tissue (2). Skeletal muscles serve to load the bone and transform skeletal segments into a system of levers; consequently, the removal of mechanical loading, as seen in periods of disuse or immobility, leads to a concurrent loss of both bone and muscle mass (3). In the craniofacial complex, the masseter muscle plays a key role in mandibular biomechanics (4). Forces generated during mastication produce significant strain, resulting in various forms of bone deformation, such as sagittal and transverse bending and twisting of the mandibular corpus (5). These forces concentrate at muscle attachment areas, including the mandibular angle and the lateral aspect of the ramus (1 , 4). Importantly, quantifying how muscle-derived mechanical loading translates into mandibular trabecular bone organization remains poorly understood in craniofacial research. On the other hand, masseter muscle thickness has been associated with masticatory function and occlusal force; however, the extent to which its morphology impacts the underlying trabecular bone organization is unclear (6 , 7). Traditional imaging methods present important limitations for assessing subtle changes in bone density and trabecular organization (8). In this context, fractal analysis (FA) was introduced as a quantitative method to describe complex trabecular patterns in radiographic images (9). FA provides an estimate of trabecular pattern complexity from radiographic images through the fractal dimension (FD), where higher values typically indicate more complex trabecular arrangements (1). In dentistry, FA has been used to assess mandibular bone pattern in periodontal disease, osteoporosis, and bone changes associated with systemic or local conditions (9). Nevertheless, despite its growing use, evidence on the relationship between muscle characteristics and bone trabecular complexity is inconsistent. Previous studies have reported heterogeneous findings, which may be partly explained by differences in imaging protocols, study populations, and control of biological variables (8). Temporomandibular disorders (TMD) are a heterogeneous group of musculoskeletal disorders that involve the temporomandibular joint (TMJ), masticatory muscles, and associated structures (10). The most common clinical manifestation of myogenous TMD is masticatory muscle myalgia (11). Myalgia can lead to chronic inflammation, resulting in increased levels of cytokines such as interleukin-6, which has been reported in the masseter muscles of adult women with myofascial pain. Considering their dual effect on bone formation/resorption, it is hypothesized that muscle-derived cytokines may be associated with mandibular bone remodeling (5). The evaluation of these musculoskeletal relationships must also account for biological variables such as sex and age, considering that sex steroids are critical regulators of bone architecture and growth (12), and bones accumulate mass until approximately age 30, after which age-dependent decreases in osteocyte lacunar density and changes in muscle thickness begin to occur (4). From a clinical perspective, clarifying the relationship between muscle morphology, inflammation, and trabecular bone structure may contribute to understanding the relationship of myogenous TMD and bone remodeling. Previous studies have evaluated trabecular bone characteristics in bruxism and other conditions using fractal analysis (1 , 12 - 16), but none have investigated whether masseter muscle morphology is associated with mandibular trabecular complexity in subjects with myogenous TMD. Therefore, the aim of this study was to evaluate the association between masseter muscle thickness and mandibular trabecular complexity in subjects with and without myogenous TMDs.

## Materials and Methods

**Figure 1:**
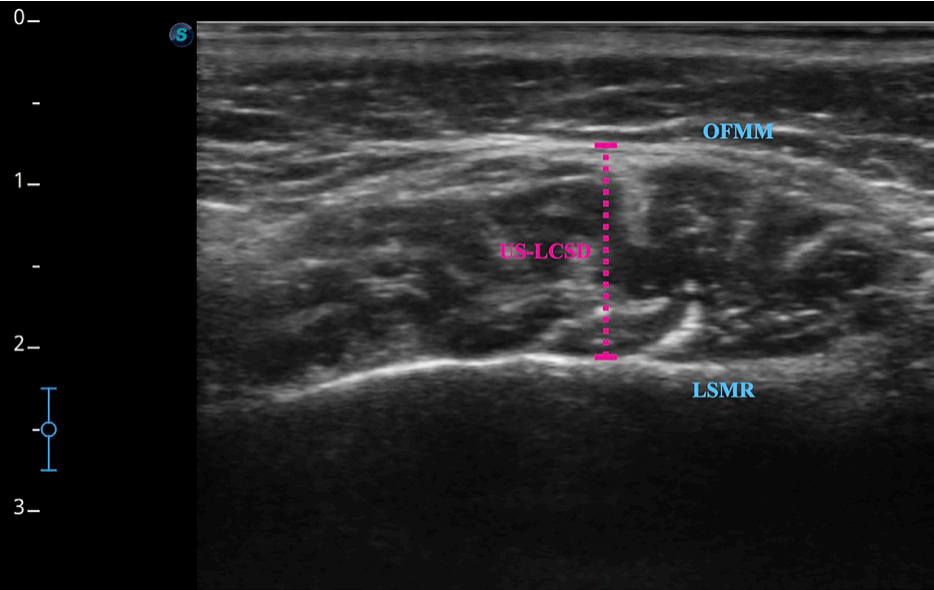
Ultrasonographic image illustrating the measurement of the local cross-sectional dimension of the masseter muscle (US-LCSD). The local cross-sectional dimension (dashed red line) was measured between the superficial fascia of the masseter muscle (OFMM) and the lateral surface of the mandibular ramus (LSMR). This measurement represents the perpendicular distance between the muscle boundaries and was used as an estimate of the masseter muscle thickness.

1. Study design A cross-sectional study was conducted at a local university dental clinic to address the research purpose. The study protocol, design, and implementation were approved by the Scientific Ethics Committee of the Faculty of Dentistry of Andrés Bello University, Viña del Mar, Chile (project no. 187-24; Apr 28, 2023). Informed consent was obtained from all the subjects included in the study. 2. Participants Participants were recruited from a university population, yielding a relatively homogeneous sample (Viña del Mar, Chile). All students were invited to participate in the study via email, accompanied by a brief explanation of the study methods. Of the 4,354 students, 115 volunteers were selected based on their responses and were invited to participate in the clinical assessment based on the DC/TMD Axis I physical examination. A diagnostic panoramic radiograph was obtained to detect possible dental pathology and used as input for estimating the trabecular complexity of the mandibular ramus. The exclusion criteria were presence of dental pathologies and/or oral/maxillofacial infections, tumors and/or cysts; ongoing orthodontic treatment; ongoing antidepressant or muscle relaxant drug therapy; presence of arthrogenous TMD. Subjects were excluded if their panoramic radiograph lacked sharpness or if signs of movement during image acquisition were detected. After the clinical and imaging assessment, subjects with a TMD diagnosis received initial management based on behavioral measures and were referred to a TMD specialist if necessary. 3. Variables and data measurements 3.1 Demographic variables. During recruitment, age (continuous variable, in years) and sex (nominal variable, as female/male) were recorded for each study subject. 3.2 Clinical condition. According to the DC/TMD axis I assessment of the masseter muscles, subjects' condition was categorized as a dichotomous variable: TMD subject (if myalgia was present) or Control subject (if myalgia was absent). Also, each side was categorized nominally as TMD or control. 3.3 Ultrasonographic Local cross-sectional dimension (US-LCSD) of masseter muscles. All subjects underwent ultrasonographic evaluation by a TMD specialist with experience in USG using a 12 MHz linear probe (E1 exp, Sonoscape Medical Corp., Guangdong, China) in B-mode and MSK preset. To avoid potential bias, subjects were identified only by their study ID number, and the examiner was blind to their clinical condition. For standardized ultrasonographic image capture, an anatomically located, fixed US probe position was used (17). During the exam, the subject was sitting in an upright position, maintaining the teeth in centric occlusion with a light occlusal contact. The US probe was positioned using minimal pressure, perpendicular to the anterior margin of the superficial masseter muscle and external surface of the mandibular ramus, between 2-2.5 cm above the lower mandibular margin. The following anatomical landmarks were considered to determine the US probe location: anterior border of the masseter muscles, lower jaw border, an anterior point located 2-2.5 cm above the lower mandibular margin over the anterior border of the masseter muscle, and a posterior point located 2-2.5 cm above the lower mandibular margin immediately anterior to the mandibular posterior border. Once located, the US probe was corrected with subtle tilting and rotation until a complete, undistorted transverse section of the masseter muscle was achieved, and the resulting image was captured for further analysis. This process was repeated three times per muscle. The US-LCSD consisted of measuring the thickness or width of the central part of the masseter muscle, defined as the maximum distance (in millimeters) between the outer fascia of the muscle and the lateral surface of the ramus (Fig. 1).

1

**Figure 2:**
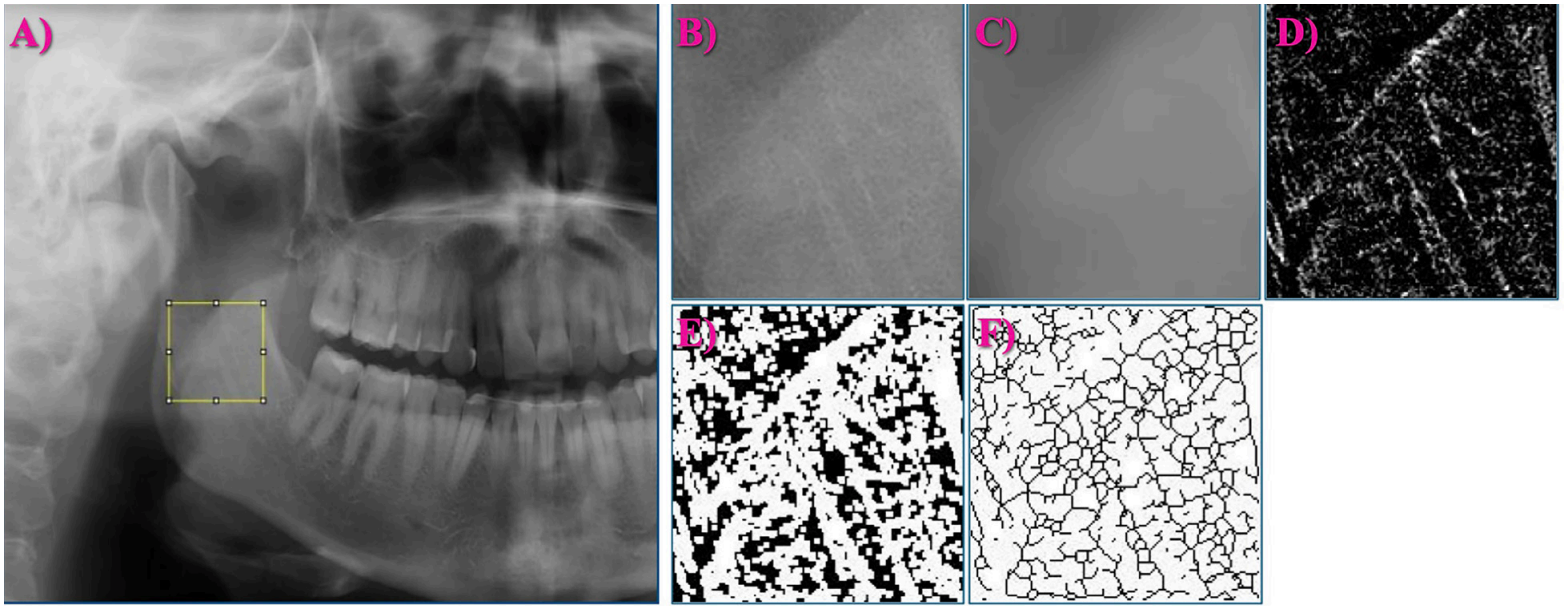
Fractal analysis workflow. Representative steps of the fractal analysis procedure applied to panoramic radiographs. (A) Selection of the region of interest (ROI) in the mandibular ramus. (B) Original ROI. (C) Gaussian-blurred image. (D) Image obtained after subtraction of the blurred image from the original. (E) Binary image after thresholding and morphological processing. (F) Skeletonized image used for fractal dimension calculation.

Finally, the US-LCSD was treated as a continuous variable per side, and as the mean US-LCSD of both sides per subject. 3.4 Fractal Dimension (FD). Standardized panoramic radiographs (73 kV, 13 mA, and 14 s) were obtained for all participants using a digital panoramic imaging system (ProMax 2D Image receptor, Planmeca, Helsinki, Finland). For image acquisition, subjects were positioned with the Frankfort horizontal plane parallel to the floor and the sagittal plane perpendicular to the floor. All radiographs were obtained by a single trained radiographic technician to ensure consistency in image acquisition. Using these radiographs, a fractal analysis was performed using ImageJ software (version 1.52; National Institutes of Health, Bethesda, MD, USA). For each radiograph, two symmetrical regions of interest (ROIs) measuring 250 × 250 pixels were selected at the geometric center of the right and left mandibular rami (Fig. 2A).

2

All ROIs were selected by the same examiner following a standardized protocol. Prior to the assessments, the observer underwent a calibration session using a separate set of radiographs not included in the sample, during which the ROI selection procedures for fractal analysis were standardized. Fractal dimension (FD) was calculated using the box-counting method described by White and Rudolph (18). Each ROI underwent the same series of image-processing steps prior to fractal computation. First, the ROI was duplicated (Fig. 2B), and a Gaussian blur filter was applied to the duplicate image (Fig. 2C). The blurred image was then subtracted from the original image to enhance trabecular structures (Fig. 2D). The resulting image was converted to a binary format using the "Make Binary" function, followed by erosion, dilation, and image inversion (Fig. 2E). Finally, the image was skeletonized to obtain the trabecular framework (Fig. 2F). Fractal dimension was calculated from the skeletonized trabecular pattern using the "Fractal Box Counter" tool located in the Analyze menu of ImageJ. The ROI was subdivided into grids of decreasing box sizes (2, 3, 4, 6, 8, 12, 16, 32, and 64 pixels). For each box size, the number of boxes containing trabecular structures was counted and divided by the total number of boxes within the ROI. These values were plotted on a logarithmic scale, and the fractal dimension was obtained from the slope of the resulting regression line. All image-processing steps were automated using a custom macro script developed for ImageJ to ensure standardized processing across all images. Finally, the FD value was treated as a continuous variable per side, and as the mean FD value per subject. 4. Study size The sample size was estimated based on differences in masseter thickness, as no prior studies were available to estimate the effect size for the primary association. Sample size estimation was performed using G*Power based on the difference in masseter muscle thickness reported by Imanimoghaddam et al. (19). Assuming a two-tailed independent-samples t test, = 0.05, statistical power of 80%, and the effect size derived from the reported means and standard deviations, the minimum required sample size was estimated at 56 participants. To account for potential exclusions during diagnostic assessment and imaging data verification, recruitment exceeded the minimum estimate. The final analyzed sample comprised 77 participants. 5. Statistical methods Statistical analyses were performed using JASP software (version 0.19.3; JASP Team, Amsterdam, Netherlands). Continuous variables were summarized using means, standard deviations, ranges, and 95% confidence intervals (95% CI). Normality was assessed using the Shapiro-Wilk test. Spearman's rank correlation was used to evaluate the association between US-LCSD and fractal dimension. Differences between TMD and control subjects were evaluated using independent-samples Student's t-tests, with Cohen's d as the effect size measure. The association between US-LCSD and fractal dimension was further examined using linear regression models. A simple linear regression model was first performed, followed by a multiple regression model adjusted for age, sex, and diagnostic group. An additional exploratory model included an interaction term (US-LCSD × diagnostic group) to assess whether the association differed between groups. Ipsilateral analyses were conducted by separately examining the relationship between muscle thickness and FD on the right and left sides. A quadratic regression model was also tested to explore potential non-linear relationships. Finally, asymmetry indices were calculated for both variables, and the association between fractal asymmetry and masseter thickness asymmetry was assessed using correlation and regression analyses. Model fit was evaluated using the coefficient of determination (R²), and variance inflation factors (VIF) were used to assess multicollinearity. A two-tailed p-value &lt; 0.05 was considered statistically significant.

## Results

The data supporting the study's findings are openly available on the Open Science Framework at https://doi.org/10.17605/OSF.IO/KZ8WG. 1. Participants and descriptive data. A total of 77 subjects were included in the study: 41 controls and 36 with TMD, with a mean age of 23.974 ± 2.595 years. Descriptive statistics for masseter muscle thickness and FD are shown in Table 1.

Table 1

**Figure 3:**
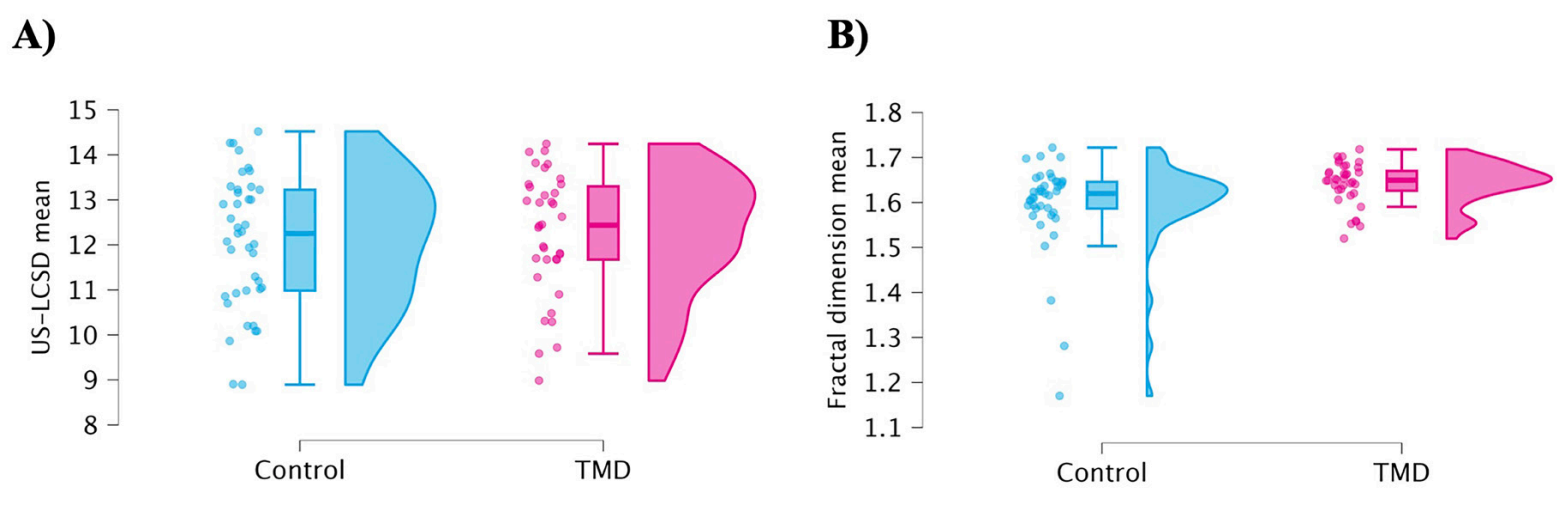
Raincloud plots showing the distribution of (A) masseter muscle thickness (US-LCSD mean) and (B) fractal dimension (mean) in control and TMD groups. Individual data points, boxplots, and kernel density distributions are presented to illustrate central tendency and variability within each group.

Comparisons of masseter thickness and FD between TMD and control subjects are shown in Fig. 3.

3

**Figure 4:**
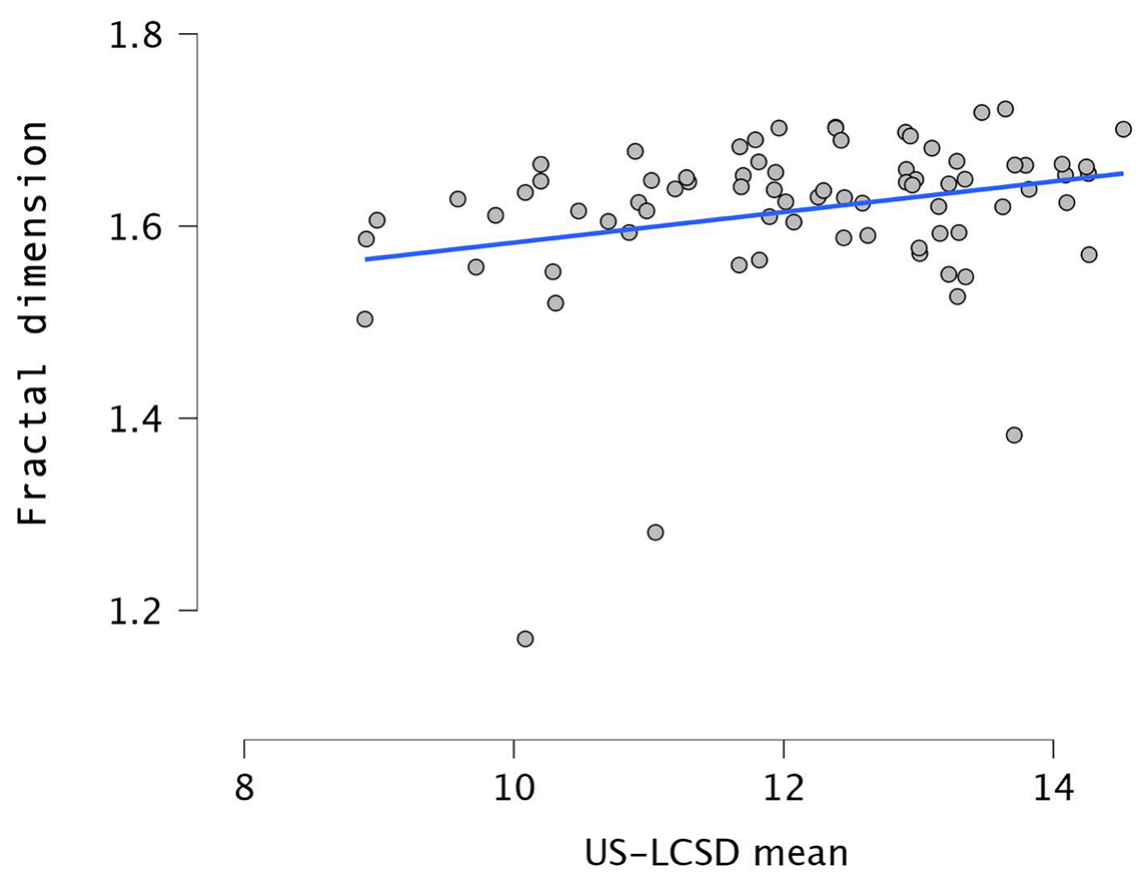
Scatter plot showing the relationship between masseter muscle thickness (US-LCSD) and fractal dimension. Each point represents an individual subject. The solid line represents the fitted linear regression model, with the shaded area indicating the 95% confidence interval.

2. Comparisons between TMD and control subjects. No significant difference in masseter thickness was observed between TMD and control groups (t = 0.597, p = 0.552; Cohen's d = 0.136). In contrast, FD was significantly higher in the TMD subjects (t = 2.491, p = 0.015; Cohen's d = 0.569), indicating a moderate effect size. 3. Sex-related differences. Masseter thickness differed significantly by sex, with higher values observed in males (p &lt; 0.001). No significant differences in fractal dimension were observed between sexes (p = 0.392). 4. Correlation and Linear Regression Analysis A weak but statistically significant positive correlation was observed between masseter thickness and FD (Spearman's = 0.255, p = 0.025) (Fig. 4).

4

In the unadjusted linear regression model, masseter thickness was significantly associated with FD ( = 0.016, 95% CI 0.003-0.029, p = 0.019), explaining approximately 7.1% of the variance (R² = 0.071). In the multivariable model adjusted for age, clinical condition, and sex, the model remained statistically significant overall (R² = 0.154, adjusted R² = 0.107, p = 0.016) (Table 2).

Table 2

However, masseter thickness was no longer significantly associated with FD ( = 0.013, 95% CI 0.002 to 0.027, p = 0.091). Age was not significantly associated with FD (p = 0.328), and sex showed no independent association (p = 0.485). In contrast, the presence of TMD remained significantly associated with higher FD values ( = 0.051, 95% CI 0.012-0.091, p = 0.011). 5. Interaction analysis The interaction term between masseter thickness and diagnostic group was not statistically significant (p = 0.836), indicating that the association between masseter thickness and FD did not differ between TMD and control subjects. 6. Ipsilateral analyses. Ipsilateral analyses showed weak associations between muscle thickness and FD on each side. On the right side, the correlation was positive but not statistically significant (r = 0.202, p = 0.078), and the corresponding regression model was not significant (R² = 0.081, p = 0.102). On the left side, a weak positive correlation was observed ( = 0.265, p = 0.020); however, the regression model did not reach statistical significance (R² = 0.095, p = 0.062). Also, no significant association was found between FD asymmetry and masseter thickness asymmetry ( = 0.189, p = 0.100). Linear regression analysis also showed no significant relationship (R² = 0.018, p = 0.246). 7. Non-linear relationship. Although the overall quadratic model reached statistical significance (p = 0.041), neither the linear (p = 0.255) nor the quadratic term (p = 0.323) were individually significant predictors.

## Discussion

The present study assessed the relationship between masseter muscle thickness and trabecular architecture of the mandibular bone using fractal analysis. A weak positive association between these variables was observed in unadjusted analyses. However, after adjustment for age, clinical condition, and sex, this association was attenuated and did not remain statistically significant. In contrast, the presence of TMD was independently associated with higher fractal dimension. The initial correlation between masseter thickness and FD is consistent with the concept of a functional relationship between masticatory muscle morphology and mandibular bone structure. However, the loss of statistical significance after adjustment suggests that this relationship is modest and influenced by underlying biological factors. Importantly, sex was strongly associated with masseter thickness but not with FD, indicating that part of the variation in muscle thickness may reflect physiological differences unrelated to trabecular bone structure. This may partly explain the attenuation of the association in the adjusted model. These results indicate that the relationship between muscle thickness and trabecular architecture is not purely direct but occurs within a broader biological context. FD has been reported to be associated with multiple biological factors, including systemic bone conditions (18 , 20), local pathological processes (21), mechanical loading (12), and demographic variables such as age and sex (12 , 18). Additionally, ongoing trabecular remodeling and physiological bone turnover may contribute to variations in FD values (12). These influences are further compounded by methodological variability inherent to fractal analysis, including differences in image processing and region-of-interest selection (8 , 9). A consistent finding of this study was that subjects with TMD exhibited higher FD compared with controls, even after adjustment for covariates. This suggests that TMD may be associated with changes in mandibular trabecular organization. Increased FD has been interpreted as greater trabecular complexity or remodeling activity in bone. It is plausible that altered functional loading, muscle activity patterns, or joint biomechanics in TMD may be related to differences in mandibular trabecular organization. Although causality cannot be inferred given the cross-sectional design, this finding supports the hypothesis that functional disturbances in the masticatory system may be associated with differences in bone microarchitecture. Previous studies have mainly examined mandibular trabecular changes in bruxism, reporting region-specific FD differences in the condyle, angulus, alveolar, or dentate regions (1 , 15 , 16 , 22), with heterogeneous findings in systematic evidence (12). In contrast, the present study specifically integrates ultrasonographic masseter thickness with fractal analysis in myogenous TMD, addressing whether muscle morphology itself relates to mandibular trabecular complexity. Although bruxism and TMD are distinct clinical conditions, evidence from studies on bruxism-one of the closest functional analogs of altered masticatory muscle activity-provides supplementary context for interpreting these findings. Previous research has shown that FD values of mandibular trabecular bone are affected by sleep bruxism, particularly at the mandibular angle and condyle (22), suggesting that repetitive muscle activity may be associated with trabecular organization. Importantly, studies incorporating clinical muscle assessment have reported that patients with bruxism and masticatory muscle pain exhibit increased FD values in specific mandibular regions (23), supporting a potential link between muscle-related dysfunction and trabecular complexity. From a biomechanical perspective, increased functional loading has been proposed to be associated with architectural modifications in mandibular bone tissue (14), consistent with adaptive remodeling processes. However, evidence remains heterogeneous, as a recent meta-analysis reported no consistent structural differences between bruxism and control subjects (12). Taken together, these findings suggest that while muscle-related functional disturbances may be associated with trabecular bone organization, such effects are likely region-specific, context-dependent, and modulated by additional biological and methodological factors. In this context, the present findings further indicate that the higher FD observed in subjects with TMD is unlikely to be explained solely by muscle morphology. Although masseter thickness showed a weak association with trabecular complexity in unadjusted analyses, this relationship did not persist after controlling for age, sex, and clinical condition, suggesting that muscle thickness alone may not be strongly associated with bone trabecular architecture after accounting for other variables. Instead, the independent association between TMD and FD supports the idea that multiple functional and pathophysiological mechanisms, such as altered loading patterns, muscle hyperactivity, joint dysfunction, or inflammatory processes, may be more closely associated with FD variation. These findings are consistent with the notion that trabecular bone characteristics may be associated with multiple biological and mechanical factors. Ipsilateral analyses revealed weak and inconsistent associations, and no significant relationship was observed between asymmetry indices. These findings suggest that the muscle-bone relationship is not strongly lateralized, supporting the use of bilateral mean values for primary analyses. From a methodological standpoint, mean FD and US-LCSD values from both sides were used to reduce individual variability and avoid potential issues related to statistical dependency. In addition, no clinical or imaging assessment of symmetry was performed, and no data on dominant masticatory side or motor preference were available. Although myogenous TMD may present unilaterally, its underlying pathophysiology and the resulting functional alterations, do not necessarily allow a clear distinction between affected and unaffected sides (24 , 25). Based on these considerations, primary analyses were conducted using bilateral mean values to represent overall muscle-bone characteristics. Additional exploratory analyses were performed separately for the right and left sides to assess potential lateralized associations. When interpreting the present results, the following limitations should be considered: (1) the cross-sectional design precludes causal inference; (2) the relatively small and homogeneous sample may limit generalizability; (3) the fractal analysis is sensitive to image processing parameters and ROI selection, and although ROI selection was standardized through examiner calibration, intra-examiner reliability was not formally quantified; and (4) the observed associations were modest, which is consistent with the multifactorial nature of bone remodeling. Given the exploratory nature of some analyses, these results should be interpreted with caution. Although the exploratory quadratic model reached statistical significance, the absence of significant individual coefficients suggests that this finding requires confirmation in larger samples. Overall, the present findings indicate that masseter muscle thickness alone is not a strong independent predictor of mandibular trabecular complexity. Instead, trabecular architecture appears to be associated with biomechanical, biological, and possibly pathological factors. From a clinical perspective, fractal analysis may capture aspects of mandibular structural variation that are associated not only with muscle morphology but also with broader functional characteristics of the stomatognathic system. Longitudinal studies incorporating functional, inflammatory, and imaging biomarkers are needed to clarify the mechanisms underlying these associations.

## Conclusions

In conclusion, a weak positive association between masseter muscle thickness and mandibular trabecular complexity was observed in unadjusted analyses but was attenuated after adjustment for demographic and clinical variables. The presence of TMD remained independently associated with higher fractal dimension values. These findings suggest that mandibular trabecular architecture may be associated with complex and interacting factors beyond muscle morphology alone.

## Figures and Tables

**Table 1:** Descriptive statistics for fractal dimension and masseter muscle thickness (US-LCSD, mm) in control and TMD groups. Values are presented as mean, 95% confidence interval (CI), standard deviation (SD), and range. Per-subject values correspond to the mean of bilateral measurements. Asymmetry between sides represents the absolute difference between right and left measurements.

	Fractal dimension				US-LCSD (in mm)			
	Per subject		Asymmetry between sides		Per subject		Asymmetry between sides	
	Control	TMD	Control	TMD	Control	TMD	Control	TMD
Mean	1.595	1.642	0.033	0.020	12.052	12.247	1.186	1.040
95% CI (lower-upper)	1.562-1.628	1.626-1.658	0.024-0.042	0.013-0.027	11.586-12.518	11.783-12.711	0.932-1.440	0.771-1.309
SD	0.104	0.048	0.029	0.021	1.477	1.372	0.806	0.794
Range	0.552	0.198	0.133	0.084	5.625	5.260	3.320	2.750

Control group (n = 41); TMD group (n = 36). Fractal analysis values are dimensionless.

**Table 2:** Linear regression analysis evaluating the association between masseter muscle thickness (US-LCSD) and fractal dimension. The model was adjusted for age, sex, and TMD.

Predictor	β (Unstandardized)	95% CI	p-value
US-LCSD mean	0.013	−0.002 to 0.027	0.091
Age	0.004	−0.004 to 0.011	0.328
TMD (vs Control)	0.051	0.012 to 0.091	0.011
Sex (Male vs Female)	0.019	−0.035 to 0.074	0.485

Model statistics: R² = 0.154; Adjusted R² = 0.107; F(4,72) = 3.287; p = 0.016.
